# Green Spaces as Healthy Places: Correlates of Urban Green Space Use in Singapore

**DOI:** 10.3390/ijerph20176711

**Published:** 2023-09-04

**Authors:** Denise Dillon, Sean T. H. Lee

**Affiliations:** School of Social and Health Sciences, James Cook University, Singapore 387380, Singapore; sean.lee@jcu.edu.au

**Keywords:** value orientations, nature connectedness, social connectedness, religious belief, spirituality, psychological wellbeing, proximity to green space, frequency of visits to green space

## Abstract

During the COVID-19 pandemic, when stress levels were heightened and social connections were threatened, a spike in green space visits was observed. Drawing upon the value–belief–norm (VBN) theory, which explains the influence of personal values and world view on perceived obligations to the environment and to action, relevant correlates were examined in relation to people’s psychological wellbeing in a bid to better elucidate this phenomenon. We aimed to explore the associations amongst a number of protective factors for psychological wellbeing and to examine the applicability of the VBN theory to wellbeing rather than environmental behaviour. Our research aim was to understand some of the correlates of the use of urban green spaces in Singapore during COVID-19. In total, 268 adult residents of Singapore completed an online survey measuring proximity/frequency of visits to green space, value orientations, nature connectedness, social connectedness, religious belief, spirituality and psychological wellbeing, along with sociodemographic variables such as age and gender. As predicted by the VBN theory, biospheric value orientation and spirituality were positively associated with nature connectedness. The nature connectedness association with psychological wellbeing was completely mediated by spirituality. Frequency of visits to nature was also positively associated with nature connectedness. Neither proximity to nature nor social connectedness were associated with nature connectedness. An altruistic value orientation was associated only with religious belief. Our results indicate that during uncertain times, people are drawing on either social or nature connections as coping mechanisms to fulfil psychological needs and enhance psychological wellbeing. Spirituality mediates this pathway for nature connectedness but not for social connectedness.

## 1. Introduction

Singapore won international praise for what was described as a national-level gold standard for detecting cases of coronavirus disease in 2019 (COVID-19) and addressing the outbreak [1]. Nonetheless, despite early assurances, by April 2020, Singapore had the highest number of confirmed cases in Southeast Asia [2]. Fear- and uncertainty-related cognitions, stress responses and coping strategies associated with the COVID-19 situation were reported internationally in the months and years since the initial detection of COVID-19 [3,4,5,6]. On a global scale, many value ideals and objects came under threat during the COVID-19 pandemic, with lockdown measures threatening to sever a level of connection with the outside world while populations were advised to remain indoors at home.

In Singapore, a circuit breaker strategy was deployed from the period 7 April to 1 June 2020. During this period, Singapore residents were advised to stay home as much as possible, to leave the house only for a limited number of sanctioned activities and to keep at least a 1 metre distance from others whenever outside the home [7]. An enforced loss of access to nearby nature for an indeterminate period was distressing to many, as was the enforced physical separation from social communities beyond those in the home environment [8]. Even with the understanding that such measures were being enforced for the greater good, individuals sought resolution through a range of coping responses. One such response was to visit green or open spaces such as parks for exercise either alone or with others they lived with, which was one of the limited number of allowed activities in Singapore while social distancing and other health safety measures were in effect [9].

As indicated by the sharp rise in visitor numbers as soon as the more severe lockdown measures eased enough to allow movement outside the home, it appears that this specific coping response was highly popular with residents despite directives to avoid unnecessary travel to parts of Singapore beyond one’s immediate neighbourhood [9,10]. Since the onset of the COVID-19 pandemic, and particularly since the lifting of more severe lockdown measures in Singapore, there has been a significant increase in individuals leaving the house to go to parks or outdoor areas also known as green spaces [11]. Given the reports of heightened stress levels amongst the population during 2020 [12,13] and research evidence indicating stress-reduction capabilities of nature immersion [14,15], it appears that people could be seeking green space visits for therapeutic purposes and for general wellbeing [16].

Even prior to the pandemic, there was some indication of people using favourite places to help self-regulate emotions and to satisfy desires or needs for pleasure, stimulation, comfort or succour [17,18,19]. However, given the spike in numbers in recent times, it appears that people might not be going out alone, and there may also be an element of belongingness or a need for social connectedness driving their coping mechanisms [20,21,22]. Indeed, there is some suggestion that emotional fulfilment of a need to relate can be satisfied through social connections to people at varying ranges of association (family, friends, acquaintances) or even through social relations with more abstract entities (my neighbourhood, my country, humanity) or to non-human animals and to nature [23]. Moreton et al. [24] went so far as to argue that “connectedness to nature may facilitate broad feelings of connectedness to humanity; it may be that a rediscovery of the wild may also lead to a renewed appreciation of the unity of humanity” (p. 64). In some ways, this large-scale nature-seeking behaviour during the pandemic might be considered a form of social movement to reclaim one’s environmental identity as part of a human species during a period of enforced social exclusion. We do not refer to committed activism or even to less intense kinds of non-activist support associated with social movements. Instead, we refer to personal or private sphere behaviours that can nonetheless serve in support of a social movement [25].

Wilson’s biophilia hypothesis suggests an innate human attraction to nature [26], and the substantial spikes in park visitor numbers in 2020 could be an instantiation of this, although a desire to satisfy a need for social connection might also have motivated visits to green spaces. While government advice was to stay at home for all non-essential activity, Singapore residents flocked to parks (e.g., Bukit Timah Nature Reserve) in what could be a form of self-therapy during uncertain times. Indeed, the large-scale and widespread nature seeking behaviour during the pandemic could be a response to fulfil a basic psychological need. Based on critical reviews of nature-relatedness literature, both Baxter and Pelletier [27] and Hurly and Walker [28] provided extensive arguments to affirm that nature relatedness meets each of Baumeister and Leary’s [29] need standards. Both groups of authors refer to Nisbet, Zelenski and Murphy’s definition of nature relatedness as “the affective, cognitive, and experiential relationship individuals have with the natural world or a subjective sense of connectedness with nature” [30]. Baxter and Pelletier elaborated the strong support for the need for nature relatedness as a need-as-motive as well as adequate support for the need as a need-as-requirement. For their part, Hurly and Walker agreed that nature relatedness is a basic psychological need but offered the caveat that natural environments do not always evoke pleasure and can instead induce fear. Hence, nature relatedness should not be assumed as a basic need in all cases or in all contexts. For instance, amongst adolescents involved in a birth cohort study, reasons for visiting green spaces included physical and social activities and relaxation, with the intention to experience nature the least reported reason [31]. As such, we should seek out other influences working in association with the need for nature relatedness during times of uncertainty.

**Figure 1:**
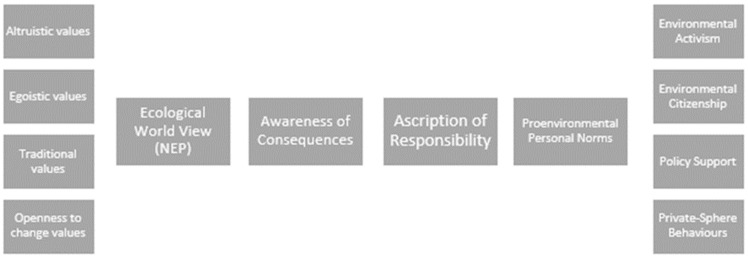
“Schematic model of variables in the Value–Belief–Norm theory as applied to environmentalism”, Reprinted with permission from Stern et al. [25]. 2022, Paul Stern. Direct causal relationships between pairs of variables operate from left to right in the model.

The value–belief–norm theory explains the influence of personal values and world view on one’s perceived obligations to the environment and further to action. Stern and Dietz [32] detailed three value bases for environmentalism: egoistic, altruistic and biospheric values. Stern et al. [25] described their value–belief–norm theory of support for social movements, as depicted in Figure 1, thusly:

*Individuals who accept a movement’s basic values believe that valued objects are threatened and believe that their actions can help restore those values experience an obligation (personal norm) for pro-movement action that creates a predisposition to provide support; the particular type of support that results is dependent on the individual’s capabilities and constraints*

(p. 81)

Value orientations have been found to explain beliefs and intentions associated with environmental behaviour [33]. In this sense, values are considered to be beliefs upon which one acts by preference or guiding principles in life, and, according to Stern and Dietz’s VBN perspective, value orientations are defined by preferences to either transcend (altruistic or biospheric orientations) or to enhance (egoistic orientation) the self [32]. These orientations might also be considered as environmental concerns oriented towards different types of valued objects: self (egoistic), other (altruistic) and the biosphere (biocentric) [34].

An ecological world view represents post-materialist values and emphasises or prioritises nature as opposed to the dominant social paradigm. While an ecological world view has been measured using the New Environmental Paradigm (NEP) [35,36], such a world view can also be conceptualised as a connectedness to nature. Connectedness to nature (CN) can be defined in various ways, but we draw on Mayer and Frantz’s view of CN as “trait levels of feeling emotionally connected to the natural world” [37], which can predict environmental behaviour and subjective wellbeing. A strong positive correlation between Mayer and Frantz’s measure of CN with the New Environmental Paradigm (NEP) scale [35] indicates good convergent validity between the two constructs. Mayer and Frantz also reported a significantly positive association between their Connectedness to Nature Scale (CNS) and a biospheric value orientation, as measured using a general value scale. The CNS was associated with neither altruistic nor egocentric values, both of which are human focused. Using just two items to measure self-transcendence values of universalism and benevolence, a study in the US found that personal experience of COVID-19 strengthened these values [38].

As for beliefs, Stern et al. [25] refer to ‘Awareness of consequences’ and ‘Ascription of Responsibility’ in their model, referring to beliefs about threats to self or others and to one’s actions towards alleviating consequences, whereas there is some indication that religious and spiritual beliefs are associated with both nature connectedness and psychological wellbeing. Research suggests a direct relationship between nature exposure and wellbeing, both physical and psychological [27,28,30,39,40]. Social connectedness is also positively associated with subjective wellbeing. Some evidence suggests that nearby nature (in the form of views from home and surrounding area) might serve as a buffer against the effects of low social connectedness [21], such that contact with nature satisfies the same underlying need for connection or belongingness as contact with people. However, it is possible that socially isolated individuals might be visiting green spaces to help satisfy their need to feel connected with other people as well as with the environment.

With respect to psychological health outcomes, evidence also indicates that several factors could either mediate or moderate this relationship. For example, spirituality has been found to mediate the nature engagement–wellbeing relationship [41], and religion has been found to be associated with health outcomes [42]. While some might argue that spiritual experiences are more likely associated with wilderness or remote natural environments that would support facets of spiritual experiences such as awe, Baur [43] reported spiritual experiences associated with built environments such as urban gardens and hospital healing gardens, which indicates that wild or remote landscapes are not an essential factor.

### The Current Study

**Figure 2:**
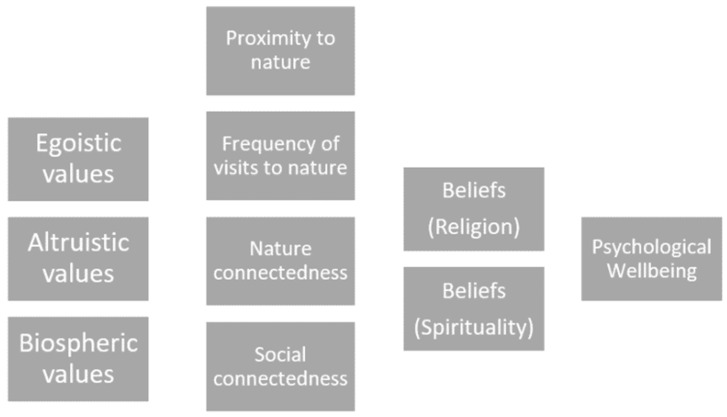
Schematic model of predictor and outcome variables.

Observations of behaviour whereby residents have been actively seeking nature experiences during uncertain times provided an opportunity to explore the associations amongst a number of protective factors for psychological wellbeing and to examine the applicability of the VBN theory to wellbeing rather than environmental behaviour, as we have modelled in Figure 2. As in Stern et al. [25] and represented in Figure 1, we propose that the direct causal relationships between pairs of variables operate from left to right in the model. An understanding of what motivates people to seek outdoor experiences in stressful times could be used to inform urban planning policies to help ensure that sufficient green spaces are accessible. Consequently, our research aim was to understand some of the correlates of the use of urban green spaces in Singapore during COVID-19. More specifically, we aimed to gain an understanding of some of the known correlates with nature connectedness and seeking behaviours of outdoor experiences in green spaces in Singapore, and how these are associated with wellbeing. Following a marked increase in individuals visiting green spaces, we wanted to see if this activity could be measured as a form of social movement (personal and private sphere behaviours) towards outdoor environments, motivated by a basic need for connectedness (with people or with nature), with people’s values and world views with respect to nature guiding their choices to visit green spaces during periods of restricted social connectivity. Additionally, we aimed to explore the effects of value orientations and world views with respect to nature connectedness and religious and spiritual beliefs on psychological wellbeing. We posed our research question as follows: What form of relationships are at play amongst protective factors for psychological wellbeing in the context of nature-seeking behaviours in Singapore?

For hypotheses, based on expectations guided by the VBN model and associated literature, we made several predictions, as follows:

**H1.**  *Biospheric values, proximity to nature, frequency of visits to nature, social connectedness and spirituality would be positive correlates of nature connectedness;*

**H2.**  *Altruistic and egoistic values would be positive correlates of religious belief;*

**H3.**  *Both nature connectedness and social connectedness would positively predict psychological wellbeing;*

**H4.**  *Religious and spiritual beliefs would mediate the association between nature connectedness and wellbeing;*

**H5.**  *Proximity to nature would moderate the positive association between social connectedness and nature connectedness.*

## 2. Materials and Methods

### 2.1. Design

A correlational design and a convenience sampling recruitment method were adopted. Specifically, an online survey was administered via Qualtrics to participants recruited through an undergraduate research participation pool consisting of James Cook University psychology students enrolled in specified subjects who were eligible for partial course credit in exchange for research participation. This recruitment was managed through the SONA participant recruitment and management platform [44]. Additional recruitment methods were social media and word-of-mouth. This helped increase demographic heterogeneity in (and, by extension, generalisability of) participants beyond undergraduate students. The survey consisted of a prefacing information section and statement of consent preceding 97 items in 7 sections. Estimated completion time was approximately 10–15 min and responses were anonymous. Data collection occurred in two phases; between February and May 2021 (enabling an undergraduate final-year thesis project with a specified submission deadline) and between June 2021 and January 2022 (with the inclusion of one additional item). Ethical approval for the study was granted by the James Cook University Human Research Ethics Committee.

### 2.2. Participants

Participants were 268 adult residents of Singapore (target age range 18–35 years; 8 respondents were older than this, up to a maximum age of 64) with a mean age of 23.85 years (*SD* = 6.56). Missing data were, by default, handled pairwise for correlation and listwise for regression analyses. Participants included students from the Singapore campus of a private university, as well as community residents (citizens, Permanent Residents, those on a valid Employment Pass). Most participants were female and most identified themselves as being religious, a quarter as spiritual but not religious and a third as being neither. Eligible students from the private university received partial course credit for their participation; other participants received no compensation.

### 2.3. Measures

A 5-item demographic questionnaire captured information about age, sex, types of belief (i.e., religious, spiritual but not religious, neither), frequency of visits to green spaces since April 2020 (the onset of the initial ‘circuit breaker’ to mitigate the spread of COVID-19 in Singapore) and proximity of home to any green space. In a second round of data collection, an additional item was added to capture reasons for visiting green spaces (see Table 1).

Value orientations were measured using de Groot and Steg’s [33] 12-item scale, wherein participants responded from −1 = “opposed to my values”, 0 = “not important”, 1 = “important” to 7 = “extremely important”, on how they felt towards specific value descriptors. The scale consisted of three subscales, each measuring a specific value orientation: egoistic (e.g., “social power”, “wealth”), altruistic (e.g., “equality”, “social justice”) and biospheric value orientation (e.g., “pollution-prevention”, “respecting the earth”). As per the developers’ stated procedures, mean average was taken for each subscale to produce three indicators, each corresponding to one of the three value orientations measured. De Groot and Steg reported a high overall internal consistency of α = 0.83.

Affective and experiential connection to nature was measured using Mayer and Frantz’s [37] 14-item Connectedness-to-Nature Scale (CNS), wherein participants were tasked to rate on a scale of 1 = “Strongly agree” to 5 = “Strongly disagree” for statements such as “Like a tree can be part of a forest, I feel embedded within the broader natural world” and “I often feel disconnected from nature” (reverse scored). As per the developers’ stated procedures, a mean average was taken to reflect one’s extent of nature connectedness. Mayer and Frantz reported high internal consistency of the measure at *α* = 0.84 and a strong positive correlation with the New Environmental Paradigm (NEP) scale [35], which indicates good convergent validity.

Social connectedness was measured using Lee, Draper and Lee’s [45] 20-item Social Connectedness Scale (revised version), wherein participants were tasked to rate on a scale of 1 = “strongly disagree” to 6 = “strongly agree” for statements such as “I feel close to people” and “I don’t feel I participate with anyone or any group” (reverse scored). As per the developers’ stated procedures, scores were summed to indicate one’s extent of social connectedness.

Religious belief was measured using the 5-item Duke University Religion Index (DUREL) developed by Koenig and Büssing [42]. The DUREL consists of three subscales; one item assessing Organizational Religious Activity (ORA; on a scale of 1 = “never” to 6 = “more than once a week”, “How often do you attend Church or other religious meetings?”), one item assessing Non-Organizational Religious Activity (NORA; on a scale of 1 = “rarely or never” to 6 = “more than once a day”, “How often do you spend time in private religious activities (i.e., prayer, meditation or Bible study)?”) and three items assessing Intrinsic Religiosity (IR; on a scale of 1 = “definitely not true” to 5 = “definitely true of me” on statements such as “My religious beliefs are what really lie behind my approach to life”). While Koenig and Büssing reported a high overall test–retest reliability (two-week interval) of 0.91 and observed relatively high internal consistencies at each time point (0.78 and 0.91, respectively), they recommended for analyses to be conducted at subscale levels, as opposed to summing all five items to produce an overall religiosity score. Accordingly, only the three items assessing IR were summed.

Spirituality was measured using the 23-item Spirituality Scale (SS) developed by Delaney [46,47], which uses a 6-point response format ranging from 1 = “Strongly Disagree” to 6 = “Strongly Agree”. The SS measures 5 key components on beliefs and self-awareness: “higher power or universal”, “intelligence”, “self-discovery”, “relationship” and “eco-awareness”. Some of the items in the scale include “I find meaning in my life experiences” and “I have a sense of purpose”. As per the developers’ stated procedures, scores were summed across all items to indicate one’s spirituality levels. It should be noted that, while scores were expected to range from 23–138, due to an error in the creation of the survey, we used a 5-point format with 3 as a “neutral” option instead. As a result, our scores ranged from 23–115.

Psychological wellbeing was measured using the 5-item scale developed by the World Health Organization in 1995 [48]. The Wellbeing Index (WHO-5) employs a 6-point scale ranging from 0 = “at no time” to 5 = “all the time”. Participants responded to statements such as “I have felt cheerful and in good spirits” and “my daily life has been filled with things that interest me”. As per the developers’ stated procedures, scores were summed and multiplied by 4, with 0 indicating the worst imaginable wellbeing and 100 indicating the best imaginable wellbeing.

## 3. Results

### 3.1. Data Preparation

Missing data points were handled pairwise for correlation and listwise for regression analyses by default in IBM SPSS v.25 (IBM Corp., Armonk, NY, USA). When checking through participants’ responses, we observed an anomalous response of “1988” for age (measured in number of years) that we presumed to be an exact birth year entry. Regardless, as this remained unverifiable due to the anonymous nature of our data collection, this data point for age was excluded from our analyses. Beyond this, no other data exclusion took place. A summary of descriptive statistics is presented in Table 2.

### 3.2. Hypothesis Testing

Normality tests run on the data revealed significant violations for all but the spirituality and wellbeing measures. Both Pearson *r* correlations [49] and regression analyses [50] are quite robust to normality deviations, but we acknowledge that Spearman’s ranked order correlation is preferred for heavy-tailed distributions or when outliers are present [51]. As such, we have included both Pearson’s *r* and Spearman’s rho values in reporting of correlations.

Bivariate correlation analyses were first conducted to examine Hypothesis 1, which proposes that nature connectedness would be significantly correlated with biospheric value orientation, proximity to nature, frequency of nature visits, social connectedness and spirituality. Results confirming three of these correlations are summarized in Table 3. Neither proximity to nature nor social connectedness was significantly associated with nature connectedness.

A separate set of bivariate correlation analyses were conducted to examine Hypothesis 2, which proposed that religious beliefs would be significantly correlated with altruistic and egoistic value orientations. Results confirming the former and disconfirming the latter are summarized in Table 4, whereby the significant association with religious belief is represented in the Intrinsic religiosity subscale rather than for the activity-oriented subscales (ORA, NORA).

To test Hypothesis 3, two linear regression analyses were conducted. The first regression model examined nature connectedness as a predictor of psychological wellbeing. The model was found to be statistically significant, *R*^2^ = 0.026, *F*(1, 222) = 5.87, *p* = 0.016, with nature connectedness observed to be a statistically significant predictor, *β* = 0.161, *p* = 0.016. The second regression model examined social connectedness as a predictor of psychological wellbeing. Similarly, the model was found to be statistically significant, *R*^2^ = 0.295, *F*(1, 217) = 90.86, *p* < 0.001, with social connectedness observed to be a statistically significant predictor, *β* = 0.543, *p* < 0.001.

Next, four mediation analyses were conducted using SPSS PROCESS (Model 4; [52]) to examine Hypothesis 4. The first three independently examined ORA, NORA and IR as mediators of the association between nature connectedness and psychological wellbeing. Step 1 revealed that nature connectedness was not significantly associated with ORA, *B* = −0.07, *t*(221) = −0.32, *p* = 0.752, 95% CI = [−0.48, 0.35], NORA, *B* = 0.01, *t*(222) = 0.03, *p* = 0.977, 95% CI = [−0.39, 0.40] or IR, *B* = 0.88, *t*(221) = 1.81, *p* = 0.072, 95% CI = [−0.08, 1.84], despite nature connectedness consistently observed to be significantly associated with psychological wellbeing in Step 2 across all three models. In corroboration, testing the proposed indirect effects using a bootstrap estimation approach with 5000 sample [53] suggested that the indirect effects examined in the three models were statistically non-significant; *B* = −0.11, SE = 0.40, 95% CI = [−1.02, 0.61], *B* = 0.01, SE = 0.47, 95% CI = [−1.02, 0.94] and *B* = 0.44, SE = 0.45, 95% CI = [−0.28, 1.47], respectively.

The fourth mediation analysis examined spirituality as a mediator of the association between nature connectedness and psychological wellbeing. In Step 1, nature connectedness was found to be significantly, positively associated with spirituality, *B* = 12.43, *t*(216) = 8.87, *p* < 0.001, 95% CI = [9.67, 15.20]. In turn, spirituality was found to be significantly, positively associated with psychological wellbeing in Step 2, *B* = 0.70, *t*(215) = 6.77, *p* < 0.001, 95% CI = [0.50, 0.90]. Notably, the complete loss of statistical significance for nature connectedness as a predictor of psychological wellbeing when spirituality was added into the model in Step 2 indicates a complete mediation. This indirect effect was tested using the same bootstrap estimation approach detailed above. Results corroborated the statistical significance of the indirect effect, *B* = 8.69, SE = 1.60, 95% CI = [5.78, 12.04].

Finally, a moderation analysis was conducted using SPSS PROCESS (Model 1; [52]), with mean centring enabled, to examine Hypothesis 5. Unsurprisingly, given the observed statistically non-significant association between social connectedness and nature connectedness (correlation analysis for Hypothesis 1), no statistically significant interaction effect was found, *B* = −0.003, *t*(214) = −0.84, *p* = 0.403, 95% CI = [−0.01, 0.004].

## 4. Discussion

Our results provide some insights into the relationships amongst protective factors for psychological wellbeing in the context of nature seeking behaviours in Singapore. As predicted by the VBN theory, biospheric value orientation and spirituality were positively associated with nature connectedness as an environmental paradigm, and frequency of visits to nature was also positively associated with nature connectedness. The finding that neither proximity to nature nor social connectedness were associated with nature connectedness needs consideration. In particular, proximity to nature was positively associated with frequency of visits to nature. This indicates that ease of access during a period when visitation beyond one’s direct neighbourhood was discouraged could have been driving some of the visitation behaviour, with more frequent visits to green spaces closer to home. However, the full mediation effect of spirituality perhaps suggests that nature connectedness is innately spiritual and transcends physicality such as whether one lives close by or whether one is able to satiate social needs through that means. Living close by facilitates ease of access (and, thereby, frequency of visits) but does not change one’s inherent level of spiritual connectedness to nature. The lack of any significant association between social connectedness and most of the other variables lends weight to an argument that an environmental paradigm predominates with respect to green space visitations during the pandemic.

Stern et al. proposed that “Religious or spiritual beliefs may be especially important because they offer an absolute standard that supersedes appeals to efficiency, practicality and expedience” (p. 86); we thereby proposed that religious beliefs would be associated with human-centric value orientations. This was not the case for our sample; however, it could be that only the intrinsic religiosity measure is applicable for the current purposes, given that the ORA and NORA scales measure behaviour rather than belief per se. There was a significant association between intrinsic religiosity and altruistic value orientation, as was expected for this specific orientation, which is arguably not only human-centric, but also other-centric, whereby altruism can extend to non-human entities including nature.

As for predictions aligning with the VBN or as guided by past research, it seems likely that people are visiting green spaces because they fulfil a psychological need associated with nature connection. Our ‘primary reasons for visiting green space’ responses lend credence to this notion, with ‘Wanting to be in nature’ the most frequent response (Table 1). At the same time, it seems that green spaces might also have helped people fulfil their need to connect with other people, given the strong positive association between nature connectedness and social connectedness. Our primary data again provide some insights here. Although ‘Socialising’ was one of the least frequent reasons given, a reasonable number indicated they visited green spaces to ‘Exercise (with others)’. However, the push to get away or to be alone seems to have been another important motivator, with a combined total of 142 responses indicating ‘Exercise (alone)’, ‘Getting away from my room/home’ or ‘Getting away from people at home’.

Both nature and social connections align with psychological wellbeing. Nonetheless, given that the mean score for psychological wellbeing is very close to mid-range in our sample, it seems that participants were feeling very much less than “the best imaginable wellbeing” as would be indicated at the highest limit on this measure. This is circumstantially relevant to the underlying context of the global pandemic and general level of uncertainty with respect to future physical health. A similar consequence of the pandemic could have affected spirituality scores. The mean of 83.35 places our sample at moderate spirituality which, according to the nursing diagnosis proposed by Delaney [46], indicates potential for spiritual distress.

The observed mediation effect of spirituality (but not religious belief) aligns with past research [41], as well as with the VBN with respect to the special importance of spiritual beliefs as a standard for setting of expectations. The positive effect of spirituality on psychological wellbeing can be experienced through a sense of connectedness to nature; it is likely that visitors to green spaces are drawing on this association in their nature-seeking behaviours.

There are both theoretical and practical implications of our findings. Firstly, with respect to our novel use of the VBN model to predict wellbeing rather than environmentalism, our findings demonstrate the extended efficacy of the model beyond its original intent. With respect to the causal predictions of the VBN, we did not test the full model, but our moderation and mediation analyses assume causal alignment through the transmission of an effect from an antecedent variable onto a consequent variable [52]. Our predictions accorded with left-to-right directionality of the VBN model, with wellbeing as our outcome variable instead of the original outcome of environmentalism in Stern et al. [25]. We outlined the theoretical basis for our predictions in alignment with the VBN model due to its applicability to the underlying behaviours—specifically, the marked increase in visits to green spaces—observed during and following the periods of lockdown during a global pandemic. Secondly, our findings confirm that the underpinnings of a biophilic response to nature (e.g., stronger connectedness to nature) promote psychological benefits in terms of wellbeing such as feeling in good spirits and feeling calm and relaxed. Thirdly, whether religious belief is a reasonable feature in our revised VBN requires further consideration, due to the unexpected lack of association with egoistic values. It could be the case that the DUREL is not the most appropriate measure of religious beliefs; this could be explored further in future studies.

Practically, it is useful to have some further understanding of the correlates and antecedents of wellbeing. Further, it is reassuring to know that a lack of ready access to nature through close proximity does not appear to diminish the strength of nature connectedness. Finally, people’s need to relate might not need to be satisfied through contact with people and, instead, it could be that feeling connected to more abstract entities (e.g., my neighbourhood, a specific green space) might suffice.

### Limitations

The study was conducted during a period of relative uncertainty during a global pandemic, which is a strength for contextualisation but a limitation in terms of potential replication or situational generalisability. Further, despite efforts to recruit a more heterogenous sample, the limited age range of participants suggests they could have been predominantly university students or those in the same social networks. This implies a limited range in education level or socioeconomic status as well. While it might be considered that cultural influences might also have affected wellbeing due to varying levels of conformity to authority, for instance, this was not a feature of our adjusted VBN model and, hence, we did not deem it important to measure ethnicity as a demographic variable. While our ‘Primary reasons for visiting green spaces’ item proved useful to help nuance some of our findings, it was added only for the second round of data collection. Moreover, because participants were able to select more than one option if equally important, it was not possible to establish a single most frequent primary reason. Delaney’s [46] Spirituality Scale is not a mainstream measure and it was developed with the provision of holistic nursing practice in mind. The measure addresses self-discovery, relationships and eco-awareness and items are worded as affirmations (e.g., I am happy about the person I have become, I respect the diversity of people, My spirituality gives me inner strength). The affirmative wording of items could lead some to perceive a bias towards optimism in the measure, but Delaney expressed an intention to provide a user-friendly format for use in “diverse patient populations” (p. 162) and the range of scores allows interpretation for those in spiritual distress through to those with high levels of spiritual wellness.

Finally, a test of the full model is desirable but that would require structural equation modelling (SEM); our current sample is not sufficient to accommodate SEM. However, we recommend this be a goal for future research using the adjusted VBN model.

## 5. Conclusions

Our results indicate that, during uncertain times, people draw on either social or nature connections as coping mechanisms to fulfil psychological needs and enhance psychological wellbeing. Spirituality mediates this pathway for nature connectedness but not for social connectedness. A biospheric value orientation is associated with the environmental paradigm, focusing on pollution prevention, respect for the earth, unity with nature and protecting the environment, whereas an egoistic orientation targets the self through power, wealth, authority and influence. In contrast, our data indicate that an altruistic orientation, focusing on equality, a world at peace, social justice and helpfulness, is more closely aligned with religious belief. This other-centric orientation might prompt a movement towards green spaces through a predisposition to provide support for others. As described by Stern et al. [25], “the particular type of support that results is dependent on the individual’s capabilities and constraints” (p. 81), so that the variability in green space visitations probably aligns with some of these dispositional factors.

## Figures and Tables

**Table 1:** Sociodemographic characteristics of participants.

Demographic Measures	Count in Category	% in Category
Gender (*n* = 237)		
Female	164	69.2
Male	69	29.1
Nonbinary	4	1.7
Belief system (*n* = 239)		
Religious	97	40.6
Spiritual but not religious	64	26.8
Neither	78	32.6
Proximity to green space from home		
Adjacent to home	82	34.3
Within easy walking distance	115	48.1
Slightly far walking distance	27	11.3
Far away from home	15	6.3
Frequency of visits to green spaces		
Never	45	18.8
On a monthly basis	100	41.8
On a weekly basis	68	28.5
Daily	26	10.9
^a^ Primary reason/s for visiting green spaces (*n* = 146)		
Socialising	35	
Exercise (with others)	47	
Exercise (alone)	54	
Regular gym/training session shifted to outdoor venue	5	
Getting away from my room/home	62	
Getting away from people at home	26	
Wanting to be in nature	74	
Other	6	

Note: *n* differs across categories, depending on the number who responded to those items. ^a^ The primary reasons item was added for the second round of data collection; hence, there were fewer participant responses for this item (from *n* = 146). Participants could select more than one option if equally important. For the 6 ‘Other’ responses, 3 indicated walking the dog and 1 each for ‘part of travelling’, ‘relaxing’ and ‘to have a walk outside and let my mind wonder’.

**Table 2:** Descriptive data and internal reliabilities for measures.

	*n*	M	*SD*	Actual Range	Potential Range	No. of Items	Cronbach’s α
Frequency of Nature Visits	235	2.31	0.90	1–4	1–4	1	
^a^ Proximity to Nature	239	3.10	0.84	1–4	1–4	1	
Egoistic Value Orientation	223	3.84	1.59	−1–7	0–7	4	0.846
Altruistic Value Orientation	226	5.71	1.42	−1–7	1–7	4	0.868
Biospheric Value Orientation	223	5.14	1.64	−1–7	−1–7	4	0.906
Nature Connectedness	226	3.34	0.55	1–5	1.29–5.00	14	0.820
Social Connectedness	227	74.22	14.46	20–120	28–102	20	0.906
Religious Belief (ORA)	235	2.57	1.73	1–6	1–6	1	
Religious Belief (NORA)	236	2.22	1.64	1–6	1–6	1	
Religious Belief (IR)	234	8.35	4.05	3–15	3–15	3	0.902
Spirituality	227	83.35	13.48	23–115	52–115	23	0.903
Psychological Wellbeing	227	51.67	19.49	0–100	0–100	5	0.884

^a^ Higher values indicate closer proximity.

**Table 3:** Correlation Test for Study Variables Associated with Nature Connectedness.

		1	2	3	4	5	6	7	8
1	Nature Connectedness	-	−0.049 (−0.038)	0.296 ** (0.279 **)	0.469 ** (0.426 **)	0.077 (0.061)	0.212 ** (0.204 **)	0.095 (0.131)	0.513 ** (0.519 **)
2	Egoistic Value Orientation		-	0.197 ** (0.155*)	0.197 ** (0.183 **)	0.010 (0.022)	−0.060 (−0.075)	0.087 (0.085)	0.054 (0.067)
3	Altruistic Value Orientation			-	0.676 ** (0.632 **)	−0.005 (−0.023)	−0.065 (−0.003)	0.002 (0.037)	0.292 ** (0.347 **)
4	Biospheric Value Orientation				-	0.063 (0.042)	0.105 (0.080)	−0.045 (−0.026)	0.257 ** (0.240 **)
5	Proximity to Nature					-	0.363 ** (0.356 **)	0.090 (0.092)	0.051 (0.026)
6	Frequency of Nature Visits						-	−0.006 (0.006)	0.203 ** (0.196 **)
7	Social Connectedness							-	0.325 ** (0.341 **)
8	Spirituality								-

** *p* < 0.01, Spearman rho values in parentheses.

**Table 4:** Correlation test for associations between religious beliefs ^a^ and value orientations.

		1	2	3	4	5	6
1	ORA	-	0.649 ** (0.658 **)	0.689 ** (0.676 **)	0.047 (0.061)	−0.003 (−0.009)	−0.171 * (−0.133 *)
2	NORA		-	0.640 ** (0.662 **)	−0.004 (0.014)	−0.007 (−0.008)	−0.128 (−0.140 *)
3	IR			-	0.132 * (0.145 *)	0.017 (0.005)	−0.018 (−0.019)
4	Altruistic				-	0.197 ** (0.155 *)	0.676 ** (0.632 **)
5	Egoistic					-	0.197 ** (−0.183 **)
6	Biospheric						-

* *p* < 0.05, ** *p* < 0.01, Spearman rho values in parentheses. ^a^ Duke University Religion Index subscales: ORA = Organizational religious activity; NORA = Non-organizational religious activity, IR = Intrinsic religiosity.

## Data Availability

Data are available in a publicly accessible repository. The data presented in this study are openly available in Research Data JCU at https://doi.org/10.25903/9wys-6813.
